# The role of neural stem cells in regulating glial scar formation and repair

**DOI:** 10.1007/s00441-021-03554-0

**Published:** 2021-11-25

**Authors:** Alexandra M. Nicaise, Andrea D’Angelo, Rosana-Bristena Ionescu, Grzegorz Krzak, Cory M. Willis, Stefano Pluchino

**Affiliations:** Department of Clinical Neurosciences and National Institute for Health Research (NIHR) Biomedical Research Centre, University of Cambridge, Cambridge, UK

**Keywords:** Neural stem cells, Cell therapies, 3D modelling, Astrocytes

## Abstract

Glial scars are a common pathological occurrence in a variety of central nervous system (CNS) diseases and injuries. They are caused after severe damage and consist of reactive glia that form a barrier around the damaged tissue that leads to a non-permissive microenvironment which prevents proper endogenous regeneration. While there are a number of therapies that are able to address some components of disease, there are none that provide regenerative properties. Within the past decade, neural stem cells (NSCs) have been heavily studied due to their potent anti-inflammatory and reparative capabilities in disease and injury. Exogenously applied NSCs have been found to aid in glial scar healing by reducing inflammation and providing cell replacement. However, endogenous NSCs have also been found to contribute to the reactive environment by different means. Further understanding how NSCs can be leveraged to aid in the resolution of the glial scar is imperative in the use of these cells as regenerative therapies. To do so, humanised 3D model systems have been developed to study the development and maintenance of the glial scar. Herein, we explore the current work on endogenous and exogenous NSCs in the glial scar as well as the novel 3D stem cell–based technologies being used to model this pathology in a dish.

## Introduction

The high complexity of the central nervous system (CNS) leads to its limited ability to recover upon damage, mainly due to the scarce regenerative potential. Available treatments aim to stop damage and alleviate symptoms; therefore, therapeutic strategies that aim to promote repair are a primary focus of many studies. Regenerative medicine holds the promise to induce repair in organs and tissues after disease or injury. One of the main lines of inquiry within regenerative medicine is how to best leverage stem cells of the brain, also called neural stem cells (NSCs), both endogenous and exogenous, as potential therapy for human neurological conditions. Unfortunately, in humans and rodents, with age, the capabilities of the endogenous NSCs decline making the repair of the brain after injury or disease extremely limited. However, an ever-increasing number of studies have identified the suitability of NSCs for engraftment to positively modulate the inflammatory environment and promote reparative programs in the injured and diseased CNS (Baker et al. 2017; Fischer et al. 2020; Peruzzotti-Jametti et al. 2018; Pluchino et al. 2003).

Typically, after injury or in disease, glial cells in the CNS become reactive in response to inflammation. Inflammation can be initiated by local infiltration of periphery born immune cells, such as T and B cells—known as *adaptive inflammation*—or triggered by the resident glial cells of the brain, including microglia and astrocytes, which is known as *innate inflammation* (Amor and Woodroofe 2014). Due to the highly heterogenous response of the CNS to injuries and diseases, the initial events triggering CNS damage may be highly variable (Adams and Gallo 2018). Here, they can involve a combination of the innate and adaptive immune responses, which will affect the cytokines and secreted factors released that influence the downstream cellular and tissue responses (Bhat and Steinman 2009). However, despite the complex inflammatory processes involved in CNS injuries and diseases, the initial response of acute inflammation acts as a protective process designed to facilitate eventual repair processes. In the CNS, glial cells form what is called a glial scar, which is a structural formation consisting of reactive glia, both astrocytes and myeloid cells, as well as a variety of other cells, that surround an area of severe tissue damage (Adams and Gallo 2018). This structure is seen in a multitude of injuries and disease, including spinal cord injury (SCI), chronic multiple sclerosis (MS) lesions, stroke, and Alzheimer’s disease (AD) (Adams and Gallo 2018). Initially, the glial scar acts as a protective mechanism, preventing the spread of damage to the healthy surrounding tissue (Silver and Miller 2004). Furthermore, there is a subset of reactive astrocytes that proliferate around the lesion which have been found to help repair the blood–brain barrier after injury (Faulkner et al. 2004). Without formation of the glial scar, there is no initiation of reparative mechanisms (Gesteira et al. 2016). However, glial scars are also associated with chronic non-resolving CNS pathology (Bradbury and Burnside 2019). This is caused by subsets of reactive astrocytes and macrophages that are neurotoxic and pro-inflammatory, the deposition of extracellular matrix (ECM) proteins, and the physical barrier formed by the glial scar itself. Together, these factors contribute to the inhibitory environment of the glial scar thereby preventing repair via hindering neuronal growth (Adams and Gallo 2018). Continued work into unravelling the underlying pathology of the glial scar will be helpful in the design of new regenerative therapeutics, such as harnessing the anti-inflammatory capabilities of NSCs. In addition, targeting specific subsets of reactive glia may prove to be beneficial in the repair of the glial scar.

Herein, we discuss how endogenous and exogenously applied NSCs have a beneficial or detrimental contribution towards the resolution of the glial scar and how the glial scar can be modelled in vitro using next-generation cellular technologies towards the development and testing of more targeted therapeutics for repair in the injured and diseased CNS.

## The pathobiology of the glial scar

CNS injuries or diseases result in multifaceted cellular and molecular responses that include the formation of a glial scar. The glial scar is loosely defined as a structural formation of reactive glia that creates a physiological barrier around the perimeter of areas with severe tissue damage and lesions. Specifically, the formation of the glial scar has been extensively studied within the context of SCI (Yang et al. 2020). However, its formation has been identified after traumatic brain injury (Yang et al. 2020), ischemic stroke (Huang et al. 2014), and numerous neurodegenerative diseases, including MS (Bribian et al. 2018) and AD (D'Ambrosi and Apolloni 2020). Therefore, understanding the dynamic role of the glial scar components and their response within different injury and disease settings is an area of growing interest. Findings from these studies will aid in identifying new targets and critical windows wherein next-generation therapies can be applied. This includes promoting endogenous stem/progenitor cell responses or applying exogenous stem cells through transplants to promote the regeneration of the damaged areas of the CNS.

**Fig. 1:**
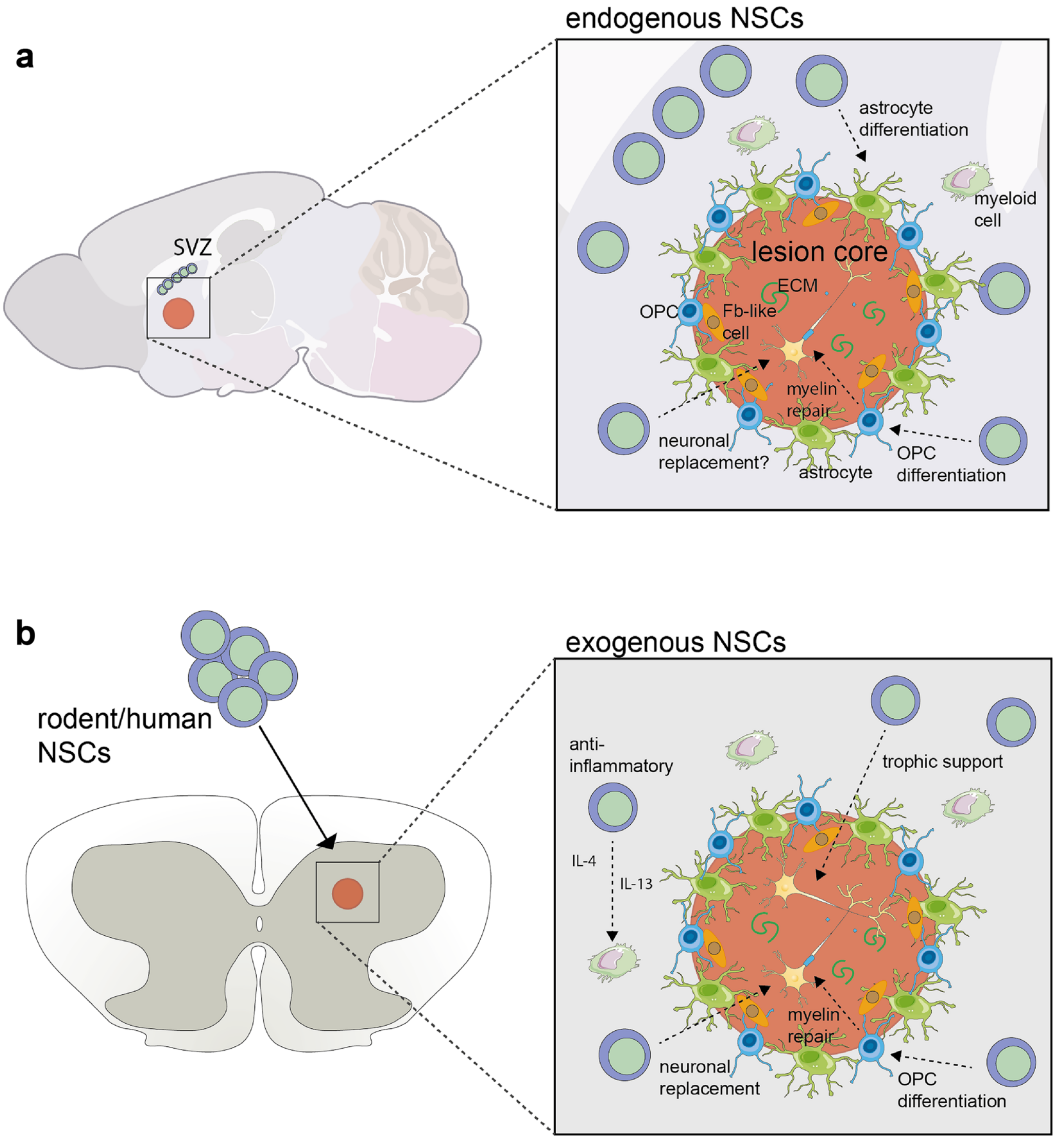
Endogenous and exogenous roles of neural stem cells in the glial scar. **A** In injury or disease, endogenous NSCs migrate to glial scar areas from stem cell niches, including the subventricular zone (SVZ) in the brain and the central canal of the spinal cord. Depending on the injury, disease, or age, NSCs can immediately initiate their proliferative response and migrate towards the site of injury, which may last up 5 weeks after injury. These NSCs have been found to contribute to reactive gliosis by differentiating into reactive astrocytes. Depending on the model of the glial scar, further studies have found that endogenous NSCs can also provide new myelination and neural replacement by differentiating into oligodendrocytes or neurons, respectively. Due to the harsh environment in the scar, including inflammation from reactive myeloid cells and astrocytes, the physical barrier formed by fibroblast-like cells (Fb-like cells), and deposits of inhibitor extracellular matrix (ECM), it is difficult for NSCs to provide total cell replacement. **B** Transplantation of exogenous NSCs from a variety of sources, including human iPS-based or from adult rodent tissue, has identified their beneficial role in amelioration of certain aspects of the scar. Herein, NSCs are anti-inflammatory and promote trophic support via the secretion of factors, including IL-4, IL-13, and NGF. Further studies have found that they can provide neuronal replacement and promote myelin repair

The glial scar comprises a highly spatio-temporal cellular heterogeneity wherein both intracellular and extracellular components contribute to its formation and its progression (Adams and Gallo 2018). Anatomically, it can be divided into two distinct cellular compartments: the lesion core and the lesion border that surrounds the core. Within the lesion core, a heterogenous mixture of cell populations exists, which includes astrocytes, fibroblast-like cells, such as pericytes and ependymal cells, and phagocytic macrophages (Yang et al. 2020). Additionally, the deposition of extracellular matrix (ECM) proteins within the lesion core, such as chondroitin sulphate proteoglycan (CSPG), forms a major inhibitory matrix. Here, these ECM proteins contribute to the inhibition of axonal regrowth which severely reduces the regenerative capacity of the glial scar and leads to further activation of pro-inflammatory myeloid cells (Gaudet and Popovich 2014). Immediately surrounding the glial scar, reactive astrocytes, NG2 glia, microglia, and other peripheral immune cells form a compact, protective border around the lesion core to prevent the spread inflammation to otherwise healthy tissue (Bradbury and Burnside 2019; Yang et al. 2020). However, over time, border-forming reactive astrocytes and macrophages are thought to become dysregulated leading to a persistent inflammatory cellular state that spreads into the surrounding healthy tissue (Fig. 1) (Bradbury and Burnside 2019).

Fundamental heterogeneity exists among glial scars due to the various ways in which a glial scar can form, as well as taking into consideration the molecular and structural variances within the CNS (Adams and Gallo 2018). Thus, the molecular architecture of the glial scar is dependent on a host of molecular and physiological contingencies, including the preceding injury or disease, the anatomical location within the CNS, the severity of the insult, and, recently, the chronological age of the individual (Adams and Gallo 2018). For example, the involvement of innate versus adaptive immune responses is known to influence the development and resolution of the glial scar. For example, in MS, the initial injury is triggered by adaptive immune responses, wherein the infiltration of peripherally activated inflammatory T cells attacks oligodendrocytes resulting in the further activation of astrocytes and microglia and then leading to the formation of a glial scar (or lesion) (Bribian et al. 2018). In the earliest stages of MS disease, the damaged areas of the CNS are partially healed (Lubetzki et al. 2020). However, as the disease progresses and the involvement of the adaptive immune system lessens, the lesioned areas are no longer capable of self-healing. This lack of resolution of the glial scar contributes to the chronic neurodegeneration observed in the latter stages of MS disease (Faissner et al. 2019). Current work has been focused on the resolution of chronic astrocyte reactivity to promote repair in the MS brain (Wheeler et al. 2020). On the one hand, MS disease is initiated by the infiltration of activated T cells into the CNS, which secrete pro-inflammatory molecules such as IFNγ and IL-17 that cause astrocyte and microglial reactivity leading to specific downstream events (Reich et al. 2018). On the other hand, SCI is initiated by a mechanical injury which causes physical damage and leads to activation of the innate immune cells of the CNS, microglia, with some involvement of peripheral macrophages (Yang et al. 2020). Astrocyte reactivity and the generation of a glial scar are therefore triggered by different mechanisms than what is seen in MS disease, including the involvement of different cell types and cytokines. Due to these inherent pathological differences, there are major disparities in the cellar mechanisms involved in the initiation of inflammation and scar formation, which should be considered when interpreting findings in the type of injury or disease setting under study.

Therefore, the extraordinary heterogeneity in reactive astroglial responses is dependent on the diversity of the stimuli encountered, as well as the pathological manifestation of the inflammation. Given this complexity, the role of NSCs is also highly variable, dependent on the injury and/or disease, and influenced by the cytokines and inflammatory mechanisms involved. Herein, we will explore the basic concepts of the glial scar and NSC interactions within various models of inflammation, such SCI, MS, and stroke.

### Cellular components of the glial scar

Astrocytes are the major player in the formation of the glial scar. The response to injury from astrocytes is called reactive gliosis, which is associated with increased proliferation, a pro-inflammatory activated state and hypertrophy. Hypertrophic astrocytes upregulate the production of intermediate filaments, such as glial fibrillary acidic protein (GFAP), nestin, and vimentin, owing to their increased size and the formation of a physical barrier around the lesion, which is believed to inhibit regeneration, along with the secretion of CSPGs. However, work has shown that astrocytes also serve a beneficial function after injury by containing the site of damage, preventing the spread to healthy tissue, which is necessary for the eventual repair of the site of injury (Faulkner et al. 2004; Gu et al. 2019). Although astrocytes are important in the early stages of scar formation to protect surrounding healthy tissue, they may also be associated with chronic progression and non-resolved inflammation, inhibiting endogenous repair. Transcriptional profiling of reactive astrocytes in mice after ischemic stroke and neuroinflammation has revealed that there are genes that can identify a pan-reactive subtype of astrocytes, termed A1-like, which produce factors that contribute to neuronal cell death (Liddelow et al. 2017; Zamanian et al. 2012). The use of binary terms to describe cells, including A1-like (pro-inflammatory) and A2-like (anti-inflammatory), has now become historically dated. Use of next-generation sequencing technologies, such as single-cell and nuclear RNA sequencing, on astrocytes has revealed the vast cellular heterogeneity of this glial in response to disease, injury, and age (Escartin et al. 2021). Moving forward, care should be taken to avoid this overly simplistic binary terminology when discussing cell-specific responses to disease and injuries.

Interestingly, the transcriptomic activation of astrocytes was found to differ based on the type of injury or disease model being used, wherein reactive astrocytes induced by ischemia demonstrated a more protective phenotype compared to an LPS astrogliosis neuroinflammation model (Zamanian et al. 2012). Despite the activation of astrocytes via systemic administration of LPS being a model of gliosis rather than glial scarring, the initiation of astrogliosis has been found to lead to the formation of a glial scar regardless (Adams and Gallo 2018).

In vivo experimental models of SCI show that A1-like reactive astrocytes populate the glial scar border and are a main component of the barrier-like structure. However, with the widespread application of RNA sequencing technologies in experimental animal models of SCI, the transcriptomic diversity of astrocytes has been recently explored ex vivo. Previous work was only able to understand bulk transcriptomic changes of specific cell types, but now, single-cell RNA sequencing (scRNAseq) has confirmed the diversity of cell subtypes in the brain. Using this technology, multiple astrocyte subpopulations have been identified after SCI, demonstrating transcriptional differences between reactive neurotoxic astrocytes (upregulation of *Nes*, *Ctnnb1*, *Axin2*, *Plaur*, *Mmp2*, and *Mmp13*) and scar-forming astrocytes (upregulation of *Cdh2*, *Sox9*, *Xylt1*, *Chst11*, *Csgalnact1*, *Acan*, *Pcan*, and *Slit2*) (Hara et al. 2017). Overall, this suggests that there are multiple distinct roles of astrocyte subpopulations in the formation and progression of the glial scar, which significantly differs based on model system. Thus, a therapeutic approach that targets specific astrocyte subtypes found to negatively influence the regenerative capacity of the glial scar may result in the greatest benefit for patients.

Myeloid cells play a key role in the formation and progression of the glial scar, as well as driving persistent neuroinflammation in the CNS (Adams and Gallo 2018). Several types of myeloid cells that contribute to the glial scar can be distinguished based on the origin of their progenitors, their anatomical localisation, surface marker expression, and cellular lifespan, which includes the CNS-resident microglia and monocyte-derived macrophages (Kierdorf et al. 2019). These cells migrate to the lesion core of the scar where they proliferate and secrete pro-inflammatory cytokines that contribute to the persistent inflammatory response. Similar to the binary classification system of astrocyte activation, myeloid cells have also been historically categorized as either pro-inflammatory, classically activated (or M1-like), or alternatively activated, anti-inflammatory (or M2-like), according to their differential gene expression (Hu et al. 2015). Just as with astrocytes, the terminology of myeloid cell activation must consider the multidimensional integration of cellular state where epigenetic, transcriptomic, proteomic, and metabolomic changes contribute to myeloid cell polarization (Ginhoux and Garel 2018; Murray 2017; Song and Colonna 2018). Therefore, in agreement with the new astrocyte nomenclature, non-binary terms should be avoided moving forward. Myeloid cells respond to proteins, nucleic acids, and metabolites released into the extracellular environment of the glial scar which are termed damage-associated molecular patterns (DAMPs) (Chen and Nunez 2010; Didangelos et al. 2016). The binding of DAMPs to their cognate receptors expressed on myeloid cells leads to their activation, resulting in morphological changes from a ramified to an ameboid shape, production and secretion of pro-inflammatory cytokines, and migration to the lesion core (Del Fresno and Sancho 2021; Pineau and Lacroix 2007). After migrating to the lesion core, activated myeloid cells have been found to impair wound healing and contribute to the inflammatory environment by persisting at the site of the injury (Nathan and Ding 2010). However, activation of microglia and macrophages renders these cells nearly indistinguishable from each other morphologically (Yamasaki et al. 2014).

Genetic strategies developed to track resident microglia in mice has revealed some of their unique qualities, such as being fundamental to the spatial organization of the glial scar (McKinsey et al. 2020; Prinz et al. 2021). Here, depletion of microglia using PLX5622, a CSF1R inhibitor, results in dysregulated glial scar formation, reduced neuronal survival, and a worsened locomotor recovery phase after SCI (Bellver-Landete et al. 2019; Zhou et al. 2020). Moreover, rather than being necessary in forming the glial scar, microglia have been found to be necessary to support remyelination of damaged nerves and promote axonal regrowth via the production of neurotrophic factors (Gaudet and Fonken 2018). In a toxin-mediated model of demyelination, endogenous remyelination was found to be dependent on the presence of anti-inflammatory ‘M2’ microglia and macrophages (Miron et al. 2013). The myeloid cell phenotype in the glial scar is ever-evolving throughout the progression of disease or after injury, having been found to shift towards a more pro-inflammatory state as the injury progresses and anti-inflammatory during repair (Milich et al. 2021). Designing ways to modify this shift and, instead, promote the anti-inflammatory, pro-regenerative functions of myeloid cells in the glial scar is an area of intense study in the field of regenerative medicine.

Fibroblast-like cells are common in the connective tissue of peripheral organs (Xu and Yao 2021). Within the CNS their location is mostly restricted to the basal laminae of the vascular system (Soderblom et al. 2013). Despite their function in the CNS under homeostatic conditions remaining mostly unknown, following injury fibroblast-like cells limit the regenerative capacity of the glial scar by sealing the lesion border, producing extracellular components that inhibit axonal regrowth, and stimulating myeloid cells to perpetuate the ongoing pro-inflammatory response (Klapka and Muller 2006). Here, reducing fibroblast-like pericyte-derived cell scarring in an in vivo SCI model resulted in less ECM deposition within the glial scar which allowed for sensorimotor functional recovery (Dias et al. 2018). Whether other, specific fibroblast-like cells contribute to glial scar formation, or if they share a common molecular marker with other CNS subtypes, such as astrocytes, is still a matter of debate.

Neuron-glial antigen 2 (NG2)^+^ oligodendrocyte progenitor cells (OPCs) have been found to rapidly react after injury in the CNS, where they proliferate and migrate to the site of injury. Along with astrocytes, they surround the lesion area, forming a physical barrier. Blocking of NG2-OPC proliferation after SCI reduces the accumulation of activated myeloid cells and reduces astrocyte hypertrophy, allowing for axonal regeneration (Rodriguez et al. 2014). This implicates NG2-OPCs in the formation and maintenance of the glial scar. Interestingly, recent work has indicated they are able to trans-differentiate into functional astrocytes, which in turn supports and maintains the architecture of the glial scar (Hackett et al. 2016). Moreover, similar to astrocytes, OPCs can become hypertrophic and overexpress (and secrete) CSPGs, thereby contributing to the inhibition of axonal regrowth and regeneration of the glial scar (Ughrin et al. 2003). On the other hand, in some instances, NG2-OPCs have also been found to undergo differentiation into mature myelinating oligodendrocytes after demyelinating injury. Herein, NG2-OPCs are given supportive cues from anti-inflammatory macrophages supporting their differentiation (Miron et al. 2013). In support of this, in a contusion SCI mouse model, OPCs have been found to differentiate into myelinating oligodendrocytes in the lesion core (Assinck et al. 2017). However, histopathological examination of some types of glial scar, such as those in human MS, has found that OPCs, rather than undergoing maturation to myelin-producing oligodendrocytes, accumulate within the lesion core and remain stuck in a progenitor state due to a yet undiscovered mechanism (Franklin and Ffrench-Constant 2017).

### The role of the extracellular matrix in the glial scar

In the glial scar, many ECM proteins that have been deposited by reactive glia, such as glycoproteins and proteoglycans, are believed to contribute to neuronal damage, chronic inflammation, and poor regenerative capacity (Bradbury and Burnside 2019). The ECM is a network of proteins that form a scaffold-like structure for cells that provides biochemical and biomechanical cues that influence cell behaviour (Barros et al. 2011). This is particularly relevant when unravelling the complex cellular interactions and signalling communications within the glial scar.

ECM proteins can be broadly divided into two groups: fibrous molecules that seal the glial scar and proteoglycans, which are extracellular molecules that can act via toll-like receptors (TLRs) to amplify pro-inflammatory responses of cells (Didangelos et al. 2016; Klapka and Muller 2006). Fibrous ECM proteins, such as collagens, act as a meshwork that can bind proteins such as semaphorins and proteoglycans that inhibit the regenerative capacity of the glial scar (Klapka and Muller 2006). In vivo, inhibition of collagen-producing pericytes results in incomplete glial scar closure, which suggests that fibrous ECMs are potential key factors in the maintenance and generation of glial scars (Goritz et al. 2011). Moreover, several other ECM proteins have been reported to inhibit axonal regeneration such as chondroitin sulfate glycosaminoglycans (Bradbury et al. 2002) and tenascin proteins (Roll and Faissner 2019). Proteomic analysis of the extracellular glial scar environment has identified several soluble endogenous alarmins. Here, soluble molecules such as the extracellular high-mobility group box-1 (HMGB1) activates pro-inflammatory IL-1β and nuclear factor kappa light chain enhancer of activated B cells (NFκΒ) signalling cascade in fibroblasts that further supports the continuation of secondary damage in the glial scar (Didangelos et al. 2016).

Interestingly, fragments of certain insoluble and fibrous ECM proteins such as tenascin, small leucine-rich repeat proteins (SLRPs), hyaluronan fragments, and sulphated proteoglycans can bind to TLRs and amplify the pro-inflammatory response (Gaudet and Popovich 2014). Thus, a therapeutic approach that inhibits the key enzymes critical for specific ECM protein biosynthesis may hold therapeutic potential in resolving the glial scar (Grimpe and Silver 2004).

## Neural stem cell interactions with the glial scar

In the adult mammalian brain and spinal cord, neural stem cells reside in neurogenic niches. Such neurogenic areas are the subventricular zone (SVZ) of the lateral wall of the lateral ventricles, the subgranular zone of the dentate gyrus (DG) of the hippocampus, and the central canal of the spinal cord (Decimo et al. 2012). Within these niches, NSCs undergo limited self-renewal and can terminally differentiate into neurons, astrocytes, and oligodendrocytes when stimulated under non-homeostatic conditions, such as in disease and injury (Llorens-Bobadilla et al. 2015; Michailidou et al. 2014). On the other hand, NSCs derived from the central canal of the spinal cord originate from ependymal stem cells, and still have the same capability of differentiating into neurons, astrocytes, and oligodendrocytes (Barnabé-Heider et al. 2010; Martens et al. 2002; Meletis et al. 2008; Sabelström et al. 2013).

In modelling pathological conditions, including MS and SCI, in rodents, previous work has demonstrated that NSCs within neurogenic niches become activated and cells can migrate into the damaged area (Butti et al. 2019; Michailidou et al. 2014; Sabelström et al. 2013). Herein, they can provide neural cell replacement in the form of astrocytes, neurons, or oligodendrocytes and aid in the regeneration of damaged tissue via the secretion of trophic and anti-inflammatory factors (Nait-Oumesmar et al. 2007; Willis et al. 2020). However, they have been found to contribute to scar formation (Stenudd et al. 2015).

### Endogenous NSC contribution to the glial scar

NSCs have been reported to play an essential function in producing protective scar-contributing astrocytes under pathological circumstances in both the brain and the spinal cord and generate a small population of oligodendrocyte progenitor cells that myelinate axons (Fig. 1A) (Barnabe-Heider et al. 2010; Grégoire et al. 2015). The earliest evidence of NSC involvement in the pathophysiological processes surrounding CNS injury was their proliferative response in the adult mouse spinal cord after SCI (Barnabe-Heider et al. 2010). Herein, it was found that a specific population of ependymal stem cells within the central canal of the spinal cord are recruited to the injury after around 2 weeks and act as bona fide NSCs, where they differentiated into astrocytes and OPCs. Two separate lineage-tracing studies examining the fate of ependymal stem cells have revealed that they exhibit multipotent traits following SCI whereby they differentiate into astrocytes and OPCs (Barnabé-Heider et al. 2010, Meletis et al. 2008). Here, the progeny of pre-labelled ependymal stem cells, astrocytes, and OPCs were also found to inhabit distinct locations following SCI 2 weeks post-injury (Barnabé-Heider et al. 2010). Whereas astrocytes gave rise to progeny that secreted growth inhibitory proteins such as CSPGs and proteoglycans and were localized to the margins of the glial scar, OPCs generated differentiated oligodendrocytes that were found distributed around the lesion core. Interestingly, ependymal stem cells differentiated not only into astrocytes, which were localized at the lesion core of the glial scar, but also into oligodendrocytes which were found within the surrounding normal appearing white matter (Meletis et al. 2008). Despite the capability to differentiate into astrocytes and oligodendrocytes, ependymal stem cells in SCI are seemingly pre-disposed towards the astrocyte lineage as they are estimated to account for approximately half of the total glial scar-associated astrocytes (Fig. 1A). They have also been reported to produce and secrete the ECM protein laminin, which is permissive for the regrowth of damaged axons (Barnabé-Heider et al. 2010; Frisén et al. 1995, Meletis et al. 2008).

In a follow-up study, SCI in adult transgenic mice with defective ependymal stem cell proliferation leads to significant defects in the formation of the glial scar, increased numbers of cleaved caspase 3–positive apoptotic neurons, and increased neuronal cell loss when compared with control mice (Sabelström et al. 2013). This work supports the idea that ependymal stem cell–derived astrocytes serve a neuroprotective role, possibly through the paracrine-mediated release of neurotrophic factors into the lesion environment. Intriguingly, there was a significant reduction in inflammatory cells within the lesion in these transgenic mice. This suggests that ependymal stem cells, rather than restricting secondary damage, are seemingly involved in the expansion of inflammatory cells within the lesion (Sabelström et al. 2013). These initial results led to the hypothesis that ependymal stem cell–derived astrocytes and reactive astrocytes are, in fact, two distinct types of glial scar–associated astrocytes that have beneficial and detrimental effects, respectively, on axonal growth and regeneration.

Similar findings were reported in a SCI model using contusion injury, which better recapitulates the pathophysiology of human SCI (Lacroix et al. 2014). In this study, the authors observed ependymal lineage stem cell proliferation occurring within the cervical spine region of mice 35 days after low thoracic SCI. These data demonstrate that the ependymal stem cell proliferative response is prolonged and can be elicited at long distance from the site of injury. It also indicates likely involvement of long-distance paracrine signalling that alters the central canal microenvironment leading to activation of the proliferative response (Lacroix et al. 2014).

However, a recent study used a genetic knock-in cell fate mapping strategy in a mouse hemisection model of SCI that found that the contribution of ependymal stem cell progeny following injury is minimal, local, and dependent on the direct injury to the ependyma (Li et al. 2016). In fact, using the same transgenic mouse, it was found that the potential of these cells for self-repair and regeneration is highly influenced by factors such as age and the lesion environment (Li et al. 2016). This was explored in juvenile mice where the induction of mild SCI, via a dorsal funiculi transection, led to the effective sealing of the lesion area by mature, endogenous glial cells rather than ependymal stem cell–derived astrocytes at 4 weeks post injury. Juvenile mice also had better recovery that was associated with decreased astrogliosis and microgliosis and reduced infiltration of pericytes and macrophages (Li et al. 2016). On the contrary, severe SCI injury in juvenile mice and any model of SCI in adult mice identified ependymal stem cells as indispensable for wound healing, acting as a reserve mechanism for self-repair when other glial cells fail to seal the lesion core (Li et al. 2016). Overall, this work highlights the important considerations of the severity of SCI and biological age when designing therapies to induce regeneration of the lesioned areas.

Intriguingly, more recent studies using mice indicate that lesion-inducing CNS injury elicits the activation, recruitment, and migration of NSCs from regions other than the traditionally defined stem cell niches to the lesion sites (Buffo et al. 2008). For instance, astrocytes in non-neurogenic regions such as the cortex and striatum have been shown to acquire neurosphere-forming capacity and generate neurons in response to pathological cues, including models of stab wound and cerebral ischemia, or to the modulation of key signalling pathways (Buffo et al. 2008; Sirko et al. 2013). Likewise, in a fate-tracing experiment, striatal astrocytes have been shown to undergo an in vivo neurogenic response up to 49 days after injury, where they differentiate into neurons, after middle cerebral artery occlusion (MCAO), an animal model of stroke, which could be recapitulated under basal conditions by blocking notch signalling (Magnusson et al. 2014).

When considering the contribution of endogenous NSCs to the formation of the glial scar, the response of NSCs within the SVZ in stroke has been the most extensively characterized. The SVZ is a highly neurogenic stem cell niche which is known to be sensitive to diffusible, proliferation-inducing factors released following brain ischemia (Grégoire et al. 2015; Lin et al. 2015; Zhang et al. 2014). Additionally, changes in the migration behaviour of NSCs have also been reported after ischemia. Here, studies using various experimental rodent stroke models, including focal cerebral ischemia and MCAO, have reported that *chains* of migrating NSCs are rerouted from the SVZ or rostral migratory stream into the ischemic zone (Arvidsson et al. 2002; Jin et al. 2003; Parent et al. 2002; Zhang et al. 2004, 2001). Interestingly, experiments performed in a mouse model of cortical ischemia have revealed that migrating NSCs default to a glial lineage and contribute to glial scar formation through a notch-dependent signalling mechanism (Benner et al. 2013). Here, targeted inhibition of notch signalling, using an inducible deletion of the Notch intracellular domain co-transcriptional activator, RBPJκ (recombination signal binding protein for immunoglobulin kappa J region), in nestin positive cells resulted in a marked shift of NSC fate in the SVZ from the astrocyte lineage towards the generation of neuroblasts which resulted in defective glial scar formation and enhanced microvascular haemorrhaging at 14 days after injury (Benner et al. 2013). Additionally, stroke-induced neurogenesis and gliogenesis have been reported to occur in the main neurogenic niche of the brain, the hippocampal dentate gyrus; however, little evidence exists that these newly formed cells are capable of migrating to other brain regions (Kernie and Parent 2010). It has also been reported that MCAO elicits a proliferative response of NSCs in ventricular zones caudal to the lateral ventricles that includes the third and fourth ventricles (Lin et al. 2015). Lastly, in addition to canonical GFAP^+^ SVZ astrocytes, ependymal stem cells of the SVZ are reported to act as an additional, but temporary, neurogenic reservoir 14 days after stroke (Zhang et al. 2007). However, these SVZ-derived ependymal stem cells are seemingly restricted to a neuronal lineage, with negligible contribution to the formation of the glial scar formation. Rather, they are rapidly depleted due to lack of the capacity for self-renewal that is retained by SVZ astrocytes (Zhang et al. 2007).

### Exogenous NSCs in treatment of the glial scar

Due to the limited pool of endogenous NSCs present in the adult, coupled with their potentially diminished regenerative potential with age, the delivery of exogenous NSCs is viewed as a promising alternative source of cells that can be delivered into the CNS to promote neurogenesis and ameliorate inflammation in CNS disorders where a glial scar is present (Chen et al. 2011; McDonald et al. 1999; Tsuji et al. 2010). Much of the interest surrounding the potential of NSC transplantation as a next-generation therapy stems from numerous, seminal studies showcasing their ability to engraft in rodents and non-human primates (Peruzzotti-Jametti et al. 2018; Pluchino et al. 2009), survive (Pluchino et al. 2003), and elicit beneficial effects via immunomodulation and trophic support irrespective of cell replacement (Willis et al. 2020).

The trophic support provided by NSC transplantation occurs via the release of soluble growth factors that act in a paracrine manner to create a supportive extracellular milieu (Fig. 1B). This prevents further degeneration of the remaining cells within the glial scar and stimulates regenerative processes (Xiao et al. 2014). These observations were gathered from numerous studies across different experimental mouse models of CNS diseases and injuries such as Parkinson’s disease, ischemic stroke, amyotrophic lateral sclerosis, and MS (Willis et al. 2020). Interestingly, in many of these studies, the engrafted NSCs preferentially accumulated within perivascular spaces of the CNS where they formed new entities termed ‘atypical niches’. Within these atypical niches, NSCs remained in an immature state; however, they were still capable of exerting immunomodulatory effects via cell-to-cell interactions with immune cells and paracrine and metabolic signalling (Fig. 1B) (Cusimano et al. 2012, Peruzzotti-Jametti et al. 2018).

Despite many earlier successes with this technology, there still remain outstanding issues centred around the therapeutic efficacy of the treatment and survival of the graft long term (Mothe et al. 2011; Tetzlaff et al. 2011). One proposed reason for this is the presence of a hostile microenvironment within the injured CNS that contains several factors, including the inflammatory environment and non-permissive ECM, that limit the survival, self-renewal, migration, and neuronal differentiation of transplanted stem cells (Charil and Filippi 2007; Dooley et al. 2014; Imitola et al. 2006; Kim et al. 2012; Neumann 2000; Singhal et al. 2008; Watanabe et al. 2007; Yiu and He 2006). Here, previous work has provided evidence in support of this hypothesis by showing that the method of cell delivery in relation to the glial scar plays a key role in graft survival and integration. Using an experimental in vivo glial scar model of the rat auditory system, Sekiya et al. challenged the dogmatic view that donor cells must be transplanted locally and demonstrated that transplantation of NSCs at the surface of the glial scar results in superior outcomes in terms of graft integration and functional recovery (Sekiya et al. 2015). These superior outcomes were attributed, unexpectedly, to the presence of the glial scar. Normally considered a challenging barrier to cell transplantation, the glial scar has been shown to harbour many important structural and chemical cues that are only preserved upon surface transplantation (Sekiya et al. 2015). For instance, endogenous astrocytes were reported to engage in the formation of a ‘glial scar bridge’ which acted as a guide to donor cells and helped support neurite elongation (Goldshmit et al. 2012). This occurred in a manner similar to that observed in CNS recovery in amphibians and fish (Goldshmit et al. 2012). Instead, it is thought that intraneural delivery either removes those cues or places the cells immediately into a hostile cellular environment that renders them unable to engage with the tissue in a beneficial manner.

A key factor in the survival of the graft is how differentiated the cells are prior to transplantation. Donor cells employed in the above study were region-restricted precursor cells at a relatively late stage of inner ear development and not bona fide neural stem cells (Sekiya et al. 2015). Indeed, NSCs expressing defined transcription factors specific to an ontogenetic stage, such as retina-specific neurons or OPC-specific, may possess a superior probability of successfully integrating into the host CNS as functional cells when compared to NSCs that have not yet begun to express such factors (MacLaren et al. 2006). In fact, NSCs isolated from various sources, such as from embryonic stem cells (ESCs) or induced pluripotent stem cells (iPSCs), and cultured in vitro for use as a regenerative therapy are inherently less neurogenic than endogenous neural stem cells and are, therefore, predisposed towards a gliogenic fate upon transplantation (Temple 2001; Zhang 2006).

There exist other components of the glial scar that render it non-conducive towards graft migration, survival, and functional integration. In particular, the group of ECM proteins known as CSPGs readily interacts with neuronal receptors that inhibit axon regeneration (Bradbury and Burnside 2019). Further, CSPGs are known to modify and enhance the neuroinflammatory processes occurring in the injured CNS (Bartus et al. 2014; Didangelos et al. 2014). Consequently, therapeutic approaches utilizing enzymatic digestion of CSPGs are a promising approach for CNS repair due to their innate ability to render the ECM more permissive to neuronal plasticity and connectivity (Suzuki et al. 2017). For example, breakdown of chondroitin sulphate-glycosaminoglycans using the chondroitinase ABC enzyme prior to NSC transplantation into the spinal cord of mice during the chronic stage of an experimental model of compression SCI led to reduced scarring, increased graft survival, and improved limb function (Suzuki et al. 2017).

Furthermore, NSCs have been found to modify the phenotype of activated myeloid cells via multiple, independent routes such as the production and release of anti-inflammatory factors, such as IL-4 and IL-13, the release and uptake of extracellular vesicles (EVs), and direct cell-to-cell contact (Fig. 1B) (Willis et al. 2020). On the one hand, in the developing brain, microglia help to support neurogenesis by regulating NSC proliferation and differentiation through the secretion of pro-inflammatory cytokines (Cunningham et al. 2013; Morton et al. 2018; Shigemoto-Mogami et al. 2014; Walton et al. 2006). On the other hand, in injury and disease, endogenous and exogenous NSCs have been found to transfer functional mitochondria that modulates the pro-inflammatory phenotype of recipient myeloid cells (Peruzzotti-Jametti et al. 2021). Although less is known about myeloid cell-NSC interactions within neuroinflammatory environments, such as the glial scar, it has been suggested that tinkering with the metabolism of pro-inflammatory myeloid cells is a novel therapeutic strategy aimed at regulating their inflammatory status (Peruzzotti-Jametti et al. 2018). Within NSCs, several mechanisms of action exist that function to modify the pro-inflammatory environment through metabolic competition for myeloid cell-derived metabolites (Pluchino et al. 2020). In particular, we have found that the intermediate metabolite of the tricarboxylic acid cycle (TCA) succinate is released from myeloid cells and accumulates extracellularly within the CSF of mice with experimental autoimmune encephalomyelitis (EAE), an experimental rodent model of MS (Peruzzotti-Jametti et al. 2018). However, intracerebroventricular injection of mouse or human NSCs into mice with EAE reduced the levels of extracellular succinate through SUCNR1 dependent and independent scavenging mechanisms and ameliorated EAE-induced pathology and associated clinical disability (Krzak et al. 2021, Peruzzotti-Jametti et al. 2018). Whether a similar scavenging mechanism exists within endogenous NSCs to limit inflammation and maintain glial scar integrity remains undetermined. However, this work provides compelling evidence that NSC transplantation could be beneficial in the resolution and regeneration of the glial scar, possibly by targeting the metabolic machinery of myeloid cells.

These studies shed light on a number of important cellular responses that could determine the feasibility and effectiveness of cell therapies for CNS repair and need to be thoroughly investigated before clinical translation can be achieved. Among these, the extent to which transplanted NSCs might potentiate reactive astrogliosis and glial scarring is of particular relevance. Understanding how to create a permissive microenvironment for exogenous NSCs and how to better facilitate their differentiation towards functional neurons and oligodendrocytes, rather than glial scar–contributing astrocytes, is important towards the development of next-generation NSC-based therapies. Furthermore, understanding how NSCs can be used as a multifaceted therapy in the treatment of injuries and disease with glial scars, such as the targeting of toxic astrocytes and inflammatory myeloid cells, may aid in the treatment of these disorders.

### Human cell sources for neural stem cell transplantation

The most important challenge in studying exogenous transplantation of NSCs is understanding how this technology can be realistically brought to clinic. To this end, the choosing of the optimal cell source with which to obtain NSCs must be evaluated. Human NSCs can be derived from multiple different sources, including foetal stem cells (FSCs) and ESCs (Liu et al. 2013), iPSCs (Rosati et al. 2018), mesenchymal stromal cells (MSCs) (Hermann et al. 2004), and directly induced NSCs (iNSCs) (Thier et al. 2012).

ESCs are pluripotent stem cells obtained from the inner cell mass of the embryonal blastocyst and are characterized by the ability to undergo unlimited self-renewal as well as the capacity to differentiate into any specialized cell type (Martello and Smith 2014). However, carrying out research on and with these cells is hampered by the ethical concerns associated with collecting these cells from aborted human embryos. Additional ethical issues are associated with using foetal NSCs due to the starting with foetal cortical tissues. In addition, ESCs and FSCs are allogeneic, and their transplantation could lead to immune rejection in the patient (Taylor et al. 2011).

With the advent of iPSC technology, the ethical and immune rejection issues associated with the other cellular sources are circumvented. These cells can be generated from the patient and have indefinite self-renewal ability and the capability to produce any type of cells of the body, including NSCs (Takahashi et al. 2007). However, concerns remain with iPS-NSCs due to their potential tumorigenic nature and genome instability after reprogramming (Desgres and Menasche 2019; Koyanagi-Aoi et al. 2013). Several groups have reported teratoma formation following the transplantation of iPS-NSCs (Itakura et al. 2017). However, even if efforts are undertaken to remove undifferentiated NSCs before transplantation, the risk of tumour formation still remains (Itakura et al. 2017).

More recently, direct reprogramming of fibroblasts into NSCs using the Yamanaka factors has been investigated. This method avoids approaches that are based on viral integration into the target genome and instead bypasses the pluripotent state in NSC generation (Thier et al. 2012). Comparing the methodology of NSC generation from iPSCs and ESCs, iNSCs can be produced faster and more efficiently than iPSC-derived NSCs and appear to be safer for transplantation, as they bypass the pluripotent state. Nevertheless, further research must be carried out on human iNSCs for their clinical application to be possible. Thus, their perceived safety could fail due to the presence of incompletely converted iNSCs within the transplanted cell preparations, or even due to the lack of genomic integrity caused by culture-driven mutations which could result in the unwanted growth of remaining or altered stem cells into tumours.

Lastly, NSCs can be differentiated from MSCs isolated from adult bone marrow. In vitro, MSCs grow as neurosphere-like structures that express neuroectodermal markers, and terminal differentiation can be obtained using neuronal or glial induction protocols (Hermann et al. 2004). Clonal analysis has shown that MSC-derived NSCs are multipotent and retain the capacity to generate both glia and neurons in vitro (Hermann et al. 2004). Moreover, they seem to also possess regenerative capabilities and immunomodulatory effects in vivo (Martin et al. 2019). However, their safety is still under study due to the risk of retaining their ability to differentiate into cells of mesodermal origin (Ullah et al. 2015).

## Modelling the glial scar using 3D stem cell technologies

Understanding the complex processes that underlie the formation of the glial scar experimental animal models is challenging not only due to the high complexity of the CNS microenvironment. From this point of view, in vitro cellular modelling systems provide a more reductionist approach that can complement findings gathered from experimental animal models. This affords researchers the unique ability to interrogate specific cellular responses and interactions in a well-defined and highly reproducible environment (Fang et al. 2019).

The complex, 3D nature of the glial scar has proven to be particularly difficult to accurately model in standard 2D in vitro systems for a number of reasons. Firstly, in vivo astrocytes have been shown to exhibit high regional, morphological, and functional heterogeneity that is unable to be fully recapitulated using in vitro 2D culture systems (Bayraktar et al. 2014; Matyash and Kettenmann 2010; Zhang and Barres 2010). These morphological differences are also reflected in the nomenclature. While in vivo astrocytes are broadly classified as either protoplasmic (with highly branched bushy processes and mainly localized in the grey matter) or fibrous (with straight and long processes and mainly located in the white matter), astrocytes in 2D culture systems are classified into type 1 (large, flat, and polygonal shaped), or type 2 (branched architecture) (Oberheim et al. 2012; Tabata 2015; Watson et al. 2017). Worth noting, the degree of resemblance between type 1/type 2 and protoplasmic/fibrous astrocytes is still unclear, and the in vitro astrocytic phenotypes are likely to represent an artefactual feature caused by their growth in rigid 2D systems. Secondly, astrocytes in vivo adopt a very complex morphology, extending intricate branched processes that interact with neurons and other cell types in three dimensions, which is not recapitulated in 2D in vitro models (Oberheim et al. 2012). Lastly, 2D in vitro cultured astrocytes are believed to adopt an artificial phenotype characterized by an artefactual activation status that can prove particularly problematic when trying to study astrocyte reactivity and the pathology of the glial scar (East et al. 2013).

Thus, the need to find more in vivo–like culture models to study the pathological mechanism of glial scar formation has led to the development and application of novel 3D culture systems, which have greatly advanced our understanding of the glial scar in vivo. Research efforts have generally focused on designing low-throughput matrices of nanofibrillar scaffolds or hydrogels that allow the investigation of the phenotypic properties of astrocytes in 3D formats (Watson et al. 2017). By using these 3D culture systems, astrocytes can be maintained in a less reactive manner than in 2D culture. This can be leveraged to induce a classical reactive response reminiscent of their behaviour in vivo upon pharmacological stimulation (East et al. 2009, 2012). Several independent studies using primary rodent astrocytes seeded into collagen-based hydrogels have reported that astrocytes were successfully established and subsequently adopted more branched, stellate, or ramified morphologies that are reminiscent of their in vivo appearance compared to the flat, polygonal morphology exhibited by astrocytes in 2D culture systems (Balasubramanian et al. 2016; East et al. 2010, 2009, 2012). The morphological features of in vitro astrocytes can further be enhanced by using collagen–hyaluronic acid hydrogels which better model the protein–glycosaminoglycan extracellular matrix environment of the brain extracellular space (Cao et al. 2012). Three-dimensional collagen gels have also been shown to be permissive not only for the differentiation of primary rodent NSCs into astrocytes but also for astrocyte migration compared to the 2D counterparts (Ge et al. 2013; O'Connor et al. 2000; Watanabe et al. 2007). This can be further enhanced by infusion of the gel with fibroblast growth factor 2 (Macaya et al. 2013).

Despite the high structural and compositional diversity of the nanofiber scaffolds and hydrogels, many shared features have resulted from their use to characterize the phenotypic properties of astrocytes in 3D formats. These include valuable insights into effects on expression of genes associated with in vivo responses to damage and disease (e.g., GFAP), as well as on cell viability, shape, and motility (Watson et al. 2017).

To allow for the study of cellular interactions in vitro, 3D astrocyte models have been further developed into complex co-culture systems with neurons or different stem cell types, including NSCs (East et al. 2010, 2009, 2013; Phillips 2014). For example, a co-culture interaction system, in which astrocytes are cultured in a gel format adjacent to gel bound primary dorsal root ganglion neurons, has been developed to study reciprocal astrocyte–neuron interactions in a 3D environment that models the axon growth inhibitory cellular interfaces that develop in the CNS in response to damage (East et al. 2012). An additional elegant 3D co-culture system has been developed by East and colleagues to assess the response of astrocytes to three cell therapies that are currently under investigation for CNS repair. The proposed model involves seeding astrocytes into 3D collagen gels which are subsequently layered on top with neural crest stem cells from hair follicles, differentiated Schwann cell-like adipose-derived stem cells, or mesenchymal stem cells from bone marrow (BM-MSCs) (East et al. 2013). In a similar fashion, a human brain endothelial cell line has been seeded on top of an astrocyte-filled collagen gel to create a 3D model of the blood–brain barrier (Hawkins et al. 2015; Sreekanthreddy et al. 2015).

It is therefore apparent that the use of different 3D culture or co-culture systems has proven to be essential for advancing our understanding on glial reactivity status and glial-scarring properties. These new culture systems have also allowed for the study if astrocyte-neural and astrocyte-stem cell graft interactions using a highly controlled and reproducible experimental setup that retains many of the in vivo properties of astrocytes. With the current developments in cerebral organoids, future work may establish their use in the study of the glial scar, as they can be maintained in vitro for several months. Furthermore, this technology lends itself to the addition of myeloid cells, which can be better studied in this 3D model (Abud et al. 2017).

## Conclusion

Rodent studies tracing endogenous NSCs in injuries and disease have revealed diversified roles for these cells depending on the model system. In SCI-based models, many endogenous NSCs have been found to contribute to astrocyte reactivity within the scar via differentiation. On the other hand, in rodent MS-model systems, endogenous NSCs contribute to repair of demyelinated areas by differentiating into myelinating OPCs. Much of this work highlights the importance of the injury or disease model system, wherein there are inherent differences in glial scar formation. This includes the location of the scar, brain or spinal cord, and exactly how the scar was formed, whether from physical injury or disease associated. Furthermore, the endogenous NSC response may be a specific mechanism seen in rodents, whereas in humans, it may be far more muted due a limited number of NSCs with age. Interestingly exogenous NSC therapy has provided much better results in the healing of the scar. Transplanted NSCs have been shown to overcome the inflammatory milieu and engraft where they provide neurotrophic and anti-inflammatory support to the damaged tissue. Nevertheless, our understanding of how NSCs can be used as a regenerative therapy is still in its infancy. Towards a better understanding of the glial scar in injury as well as disease, 3D stem cell model systems have been developed. Herein, human stem cells have been able to be differentiated into a multitude of cells found within the scar, allowing for a more targeted in vitro analysis on the exact structure and molecular makeup of this pathology, including inhibitor ECM proteins on scaffolds. Furthermore, this will help parse apart the exact mechanisms on how NSCs interact with the glial scar components, allowing for more targeted regenerative therapies.
